# Ecosystem size predicts eco‐morphological variability in a postglacial diversification

**DOI:** 10.1002/ece3.3013

**Published:** 2017-06-15

**Authors:** Hans Recknagel, Oliver E. Hooker, Colin E. Adams, Kathryn R. Elmer

**Affiliations:** ^1^ Institute of Biodiversity Animal Health & Comparative Medicine College of Medical, Veterinary & Life Sciences University of Glasgow Glasgow UK; ^2^ PR Statistics Glasgow UK

**Keywords:** adaptive morphology, Arctic charr, benthic–limnetic, ecological opportunity, environmental heterogeneity, freshwater fish, trophic morphology

## Abstract

Identifying the processes by which new phenotypes and species emerge has been a long‐standing effort in evolutionary biology. Young adaptive radiations provide a model to study patterns of morphological and ecological diversification in environmental context. Here, we use the recent radiation (ca. 12k years old) of the freshwater fish Arctic charr (*Salvelinus alpinus*) to identify abiotic and biotic environmental factors associated with adaptive morphological variation. Arctic charr are exceptionally diverse, and in postglacial lakes there is strong evidence of repeated parallel evolution of similar morphologies associated with foraging. We measured head depth (a trait reflecting general eco‐morphology and foraging ecology) of 1,091 individuals across 30 lake populations to test whether fish morphological variation was associated with lake bathymetry and/or ecological parameters. Across populations, we found a significant relationship between the variation in head depth of the charr and abiotic environmental characteristics: positively with ecosystem size (i.e., lake volume, surface area, depth) and negatively with the amount of littoral zone. In addition, extremely robust‐headed phenotypes tended to be associated with larger and deeper lakes. We identified no influence of co‐existing biotic community on Arctic charr trophic morphology. This study evidences the role of the extrinsic environment as a facilitator of rapid eco‐morphological diversification.

## 1 INTRODUCTION

Identifying the environmental agents of natural selection has proven difficult because organisms live in environments that are profoundly complex, with multiple and potentially conflicting selection pressures. Some lineages, but not all, diversify rapidly in new environments, suggesting that a combination of extrinsic and intrinsic factors determine adaptive potential (Elmer, Lehtonen, Fan, & Meyer, 2013; Losos & Mahler, 2010; Stein, Gerstner, & Kreft, 2014). It is increasingly recognized that phenotypic change can arise surprisingly fast. This has been proven experimentally (Blount, Borland, & Lenski, 2008; Kawecki et al., 2012), through artificial selection such as crop modification and animal breeding (Conner, 2003; Meyer, DuVal, & Jensen, 2012; Neff & Rine, 2006), and shown in some naturally occurring populations as a response to diversifying selection (Elmer, Lehtonen, Kautt, Harrod, & Meyer, 2010; Franks, Sim, & Weis, 2007; Hendry, Nosil, & Rieseberg, 2007).

Rapid adaptive radiations across isolated islands and lakes are well recognized as important models for disentangling how diversity arises in nature, as they provide relatively simple replicated environments in which similar phenotypic diversity has arisen repeatedly (Elmer & Meyer, 2011; Gavrilets & Losos, 2009; Schluter, 2000). Some of the best known examples include Darwin's finches on the Galapagos Islands (Grant & Grant, 2011), Hawaiian silverswords (Baldwin, 1997), *Anolis* lizards on Caribbean islands (Losos, 2009), cichlid fishes inhabiting the Great African Rift (Turner, 2007) and Central American crater lakes (Recknagel, Elmer, & Meyer, 2014), and some northern postglacial fishes (Schluter, 2000).

Colonization and adaptation in postglacial lakes has occurred relatively recently, since the last retreat of the glaciers. Postglacial lakes can be characterized as rather simple, scalable, low productivity environments (Klemetsen, 2010). They usually support habitats comprising different foraging opportunities; for example, the littoral, a shallow water zone supporting relatively high benthic invertebrate productivity, and a limnetic zone supporting planktonic invertebrate production (Robinson & Wilson, 1994; Schluter, 2000). In addition to the spatial divergence of these foraging resources, the fishes also differ in characteristics related to how they access prey: benthivorous, planktivorous, and piscivorous fishes of many species differ substantially in morphologically functional traits (e.g., Schluter, 1993; Jonsson & Jonsson, 2001; Svanbäck & Eklöv, 2004; Kahilainen & Østbye, 2006; Fraser, Huntingford, & Adams, 2008; Garduño‐Paz & Adams 2010; Willacker, von Hippel, Wilton, & Walton, 2010). These traits have been well studied and are closely related to the different foraging strategies used in the respective habitats. Fishes inhabiting littoral benthic habitats have a diet consisting of macro‐invertebrates and are usually deeper bodied, with fewer gill rakers and a more robust head. In contrast, individuals tending to utilize the limnetic environment feed on plankton and are more elongate in body shape, have a higher number of gill rakers, and more slender heads (Jonsson & Jonsson, 2001; McPhail, 1994; Østbye et al., 2006). Postglacial fishes frequently show convergence and parallelisms in trophic morphology both within and across species (Elmer & Meyer, 2011; Schluter, 2000; Seehausen & Wagner, 2014). The most prominent examples of radiating postglacial fishes include threespine sticklebacks (*Gasterosteus aculeatus*) (McPhail, 1994), European and lake whitefish (*Coregonus* sp.) (Bernatchez et al., 2010; Østbye et al., 2006), lake trout (*Salvelinus namaycush*) (Chavarie, Howland, & Tonn, 2013; Muir, Hansen, Bronte, & Krueger, 2016), and Arctic charr (*Salvelinus alpinus*) (Garduño‐Paz, Adams, Verspoor, Knox, & Harrod, 2012; Jonsson & Jonsson, 2001).

Arctic charr in particular are regarded as one of the most variable vertebrates (Klemetsen, 2013). When colonizing lakes throughout the northern hemisphere, populations have diversified dramatically across lakes that differ in their bathymetry, surface area, and ecological features (Bush & Adams, 2007; Garduño‐Paz, Demetriou, & Adams, 2010; Garduño‐Paz et al., 2012; Woods et al., 2013). Across its geographical range, Arctic charr has repeatedly evolved discrete feeding specialists (Jonsson & Jonsson, 2001; Klemetsen, 2010; Kristjánsson et al., 2011; Schluter, 2000). These trophic morphs are associated with pronounced differences in body shape. Typically, littoral macro‐benthos feeding specialists have deeper bodies and express larger, more robust heads, with a blunt snout. In contrast, plankton feeding specialists tend to have a more delicate body and head shape with smaller terminal mouths, finer jaw structure, and usually larger eyes (Adams et al., 1998; Jonsson & Jonsson, 2001; Klemetsen et al., 2003; Knudsen, Amundsen, Klemetsen, & Soerensen, 2007; Skúlason, Snorrason, & Jonsson, 1999; Snorrason et al., 1994). Morphological differences between trophic morphs are functionally linked to alternative feeding strategies that are defined by attributes of the prey (Garduño‐Paz & Adams, 2010; Hooker et al., 2016; Malmquist, 1992). In particular, larger mouth gape allows feeding on larger prey, a link that has been experimentally shown in trophically polymorphic Arctic charr (Adams & Huntingford, 2002b). Freshwater fishes in general exhibit a strong functional link between gape size, head depth, and feeding strategy (Day & McPhail, 1996; Knudsen et al., 2007; Rüber & Adams, 2001). For example, in the famously trophically diverse Icelandic Arctic charr in Thingvallavatn a primary differentiation is in head morphology where all four morphs vary significantly in head shape traits and this is associated with specialization and segregation in diet (Snorrason et al., 1994).

Research to date on adaptive radiations has suggested that ecosystem size, ecological opportunity (e.g., number of available ecological niches), and intrinsic factors (such as phylogenetic constraints and sexual selection) are vital components of diversification (Elmer et al., 2013; Gavrilets & Losos, 2009; Losos, 2010; Wagner, Harmon, & Seehausen, 2012). However, these are relatively general drivers of adaptive radiation that are likely to manifest in different specific ways in different diverging lineages. For example, the size of the habitat occupied (e.g., the area of an island or lake) may increase ecological opportunity and has been shown to predict phenotypic and species diversity (Kisel & Barraclough, 2010; Losos & Schluter, 2000; Nosil & Reimchen, 2005; Ricklefs, 2007; Seehausen, 2006). Other studies on fishes found that lake depth is a better predictor for phenotypic diversification, particularly along the benthic–limnetic axis, for example in cichlid fishes (Recknagel et al., 2014; Wagner et al., 2012) or the radiations of postglacial fishes such as sticklebacks (Willacker et al., 2010), whitefishes (Vonlanthen et al., 2009), and Arctic charr (Alekseyev, Samusenok, Matveev, & Pichugin, 2002; Hindar & Jonsson, 1982). Bathymetric traits are often correlated because lakes that increase in area also tend to become deeper and therefore more voluminous (Gavrilets & Losos, 2009; Post, Pace, & Hairston, 2000). Therefore, bathymetric traits such as lake volume and surface area are most effectively summarized as ecosystem size (e.g., Fukami, 2004; Post et al., 2000; Reche, Pulido‐Villena, Morales‐Baquero, & Casamayor, 2005). In addition, research on fishes has shown that the biotic community, such as intraspecific abundance levels (Bolnick, 2004; Svanbäck & Bolnick, 2007) and interspecific interactions such as the number of competing species (Bourke, Magnan, & Rodríguez, 1999; Robinson, Wilson, Margosian, & Lotito, 1993; Vamosi, 2003) and predators (Vamosi, 2005), also influence diversification.

Identifying the underlying factor(s) driving the repeated diversification of postglacial fishes is crucial to help understand how distinct phenotypes evolve and to predict evolutionary outcomes across environmental scenarios. Using a large dataset of Arctic charr populations from postglacial lakes across the British Isles, we tested whether an ecologically relevant morphological character—relative head depth—is correlated with the bathymetric and ecological characteristics of lakes. Head depth is closely linked to functional feeding strategy and therefore directly and indirectly reflects the extensive ecomorphological variation of Arctic charr (Adams & Huntingford, 2002a,b; Adams, Woltering, & Alexander, 2003; Jonsson & Jonsson, 2001; Liem, 1993). We examined whether the average, extremes, and extent of variation in head morphology of a charr population could be predicted by the lake environment biotic and abiotic characteristics.

## 2 METHODS

**Figure 1:**
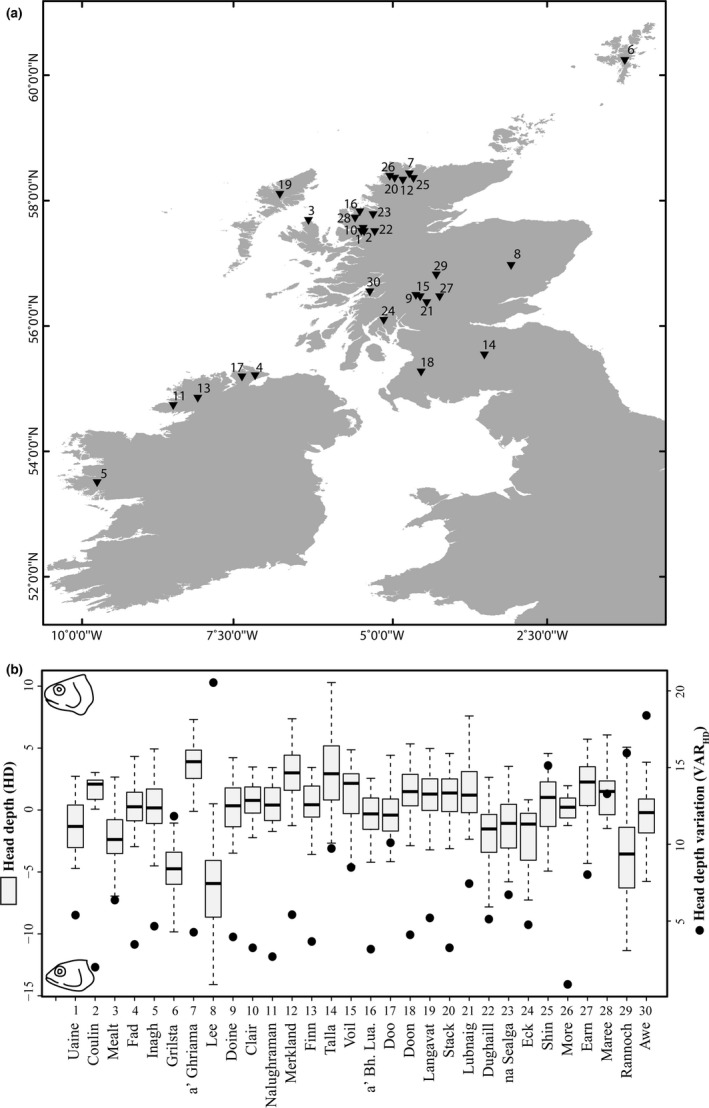
(a) Sampling sites of Arctic charr from the British Isles. Lakes are ranked according to their score on PC1 (or based on surface area in case they could not be included in the PCA), with low numbers indicating small, shallow, and species‐poor lakes while large numbers indicate greater surface area, deeper, and more species‐rich lakes. Lake names are listed with associated number in panel b. (b) Distribution of head depth is shown on the left *y*‐axis in gray boxes (black bar within box* *=* *median; whiskers* *=* *± 1.5 IQR [outliers excluded for visualization]) and variation in head depth (VAR_HD_) on the right *y*‐axis (black dots) for all individuals and across all lakes (total *N* = 1,091). Abbreviations: a’ Bh. Lua.* *=* *a’ Bhaid Luachraich

Arctic charr (*S. alpinus*) were collected from across 30 lakes in Scotland and Ireland using Nordic survey gill nets (fish total *N* = 1,091; mean 36 fish per lake, range 10–82) (Figure 1a, Table S1). Gill nets consisted of 12 panels of differently sized mesh (5–55 mm knot to knot), 30 m long and 1.5 or 6 m deep, and are nonselective for Arctic charr in the size range of 45–495 mm (fork length) (Jensen & Hesthagen, 1996). A structured random sampling approach was used to ensure that all habitats within a lake were sampled (see Adams et al., 2006 for details); nets were set in the littoral, sublittoral, profundal, and pelagic zones (*n* = 4–14 nets per lake depending upon size).

Head length, head depth, and fork length were measured for each adult individual collected. To correct for allometric effects, head depth and head length were regressed against fork length and we calculated the difference between the residuals in head depth and length for each individual; thus, a high value indicates a relatively deep‐headed individual, whereas a small value describes an individual with a shallow head relative to length. This univariate measure we use as an index for head depth (called HD hereafter) effectively describes functional ecomorphology (Adams et al., 1998) while minimizing data complexity. We calculated four morphological measures for each charr population: the average (MEAN_HD_), maximum (MAX_HD_), and minimum (MIN_HD_) head depth, and variance in head depth (Var_HD_) as a measure of morphological variation. All statistical analyses were carried out using R 3.0.2 (R Core Team 2013).

Alternatively, morphological metrics—in particular morphological variability—can be driven by demographic factors such that lakes with more individuals might exhibit greater variation in body shape, including head depth. We tested whether each of the four head depth measures depended on allelic richness, a genetic proxy for genetic diversity and population size. Estimates of microsatellite allelic richness were extracted from published literature (Wilson et al., 2004; six polymorphic nuclear microsatellite loci) on Arctic charr for 21 of the 30 lakes used in this present study (Table S1). In addition, we tested whether charr abundance had an effect on morphological traits. Abundance is an indicator for intraspecific competition within lakes and was recorded as catch per unit effort (CPUE), counted as the number of Arctic charr individuals caught per 100 m^2^ of net per 24 hr.

Environmental data were collated for each lake from published literature (Murray & Pullar, 1910), government agencies, and the authors’ own surveys (Table S1). The bathymetric parameters available were lake volume, average and maximum lake depth, lake surface area, and littoral zone area (defined as the area of the lake shallower than 4.5 m depth). The relevant biotic community parameters available were the number of competing species, number of predators, and total number of fish species in the lake community. Categorization as competitors or predators was based on the species’ ecology at adult stage. Competing species included: brown trout (*Salmo trutta*), Atlantic salmon (*Salmo salar*), roach (*Rutilus rutilus*), rainbow trout (*Oncorhynchus mykiss*), and powan (*Coregonus lavaretus*), fishes scored as predators were Northern pike (*Esox lucius*), European eel (*Anguilla anguilla*), European perch (*Perca fluviatilis*), and brown trout (*Salmo trutta*). Note that brown trout was both included as competitor and predator. European flounder (*Platichthys flesus*), brook lamprey (*Lampetra planeri*), minnow (*Phoxinus phoxinus*), and three‐spined stickleback (*Gasterosteus aculeatus*) were also included as part of the overall lake community (Table S1). Bathymetric parameters were normalized (log‐transformed). The percent of the lake substrate area categorized as littoral zone was arcsine transformed (Crawley, 2014). Ecological parameters (“biotic community”) were normalized using square root transformation (Crawley, 2014).

We used multiple lines of analysis to identify the associations among environmental characteristics and between those environmental characteristics and charr morphological variables MEAN_HD_, MAX_HD_, MIN_HD_, and VAR_HD_.

First, we inferred whether predictor variables were correlated using Pearson's correlation coefficient (PCC) and principal component analysis (PCA). Based on those correlation estimates, we assigned predictors into three separate classes as described by the first three principal components of the PCA, each sharing a set of highly correlated variables: i) PC1 represents ecosystem size, with all lake size parameters (volume, surface area, maximum depth, and mean depth) as well as biotic community size having high positive loadings on this PC (thus high PC1 scores equate to large lakes); ii) PC2 mainly describes the biotic community with high negative loadings (thus high PC2 scores define lakes with depauperate biotic communities); and, iii) PC3 is associated with small lakes that have a small littoral area (thus high PC3 scores describe small, deep lakes with a steep shore gradient) (Tables S2 and S3, Figure S1).

Second, we performed multiple linear regressions to assess the relative contribution of predictor variables to each of the four morphological variables. Models were simplified by sequentially excluding nonsignificant parameters. The full model included five predictor variables: the first three principal components representing environmental and biotic lake characteristics, genetic diversity, and charr abundance (CPUE).

As an alternative approach to address multicollinearity, we used the relative importance test to assess the contribution of the individual lake characteristic variables, ranking them by model importance and accounting for collinearity between these correlated variables to estimate their relative importance (% of *R* ^2^) (Figure S2). The relative importance test “lmg” was used within the R package relaimpo, which averages sequential sums of squares over all orderings of predictor variables (Grömping, 2006). All four morphological variables were tested against all eight correlated predictor variables by implementing a bootstrapping algorithm (*N* = 1,000) to assess 95% confidence intervals.

## 3 RESULTS

### 3.1 Relationship among environmental variables

A number of lake environment characteristics were related, as inferred from the correlation estimates (PCCs; Table S2). Bathymetry traits (lake volume, surface area, maximum depth, and mean depth) were highly correlated. Proportion of littoral zone—an important feeding habitat for fishes—was negatively correlated with mean and maximum lake depth, showing that shallower lakes had relatively more littoral zone. Biotic community variables (fish community size, number of competing species, and number of predators) were highly correlated with each other and to a lesser extent also with ecosystem size or amount of littoral zone (Table S2).

PCA drew out similar relationships among environmental variables, with the amount of littoral zone loading in the opposite direction from ecosystem size on PC1, and biotic community distinct from bathymetric variables on PC2 (Figure S1). Overall PC1 captured a high proportion of variance (loadings all exceeded 0.3) for all abiotic and biotic variables. PC3 scores increased for lakes with a small surface area and small littoral zone but high volume (i.e., deep lakes relative to surface area). Overall, the first three axes explained 89.3% of the total variance (Table S3).

### 3.2 Eco‐morphology across populations

The Arctic charr populations differed dramatically in their head depth across lakes (Figure 1b). The most slender‐headed individual came from Loch Lee (MIN_HD_ of −16.91), and the individual with the bulkiest (deepest) heads were from Loch Awe (MAX_HD_ of 25.45). MEAN_HD_ varied across lakes, from −7.06 in Loch Lee to 3.72 in a’ Ghriama.

A considerable number of Arctic charr populations showed high variability in head morphologies across lakes, with the most variability found in the populations of Loch Lee (VAR_HD_ of 20.54), followed by Loch Awe (VAR_HD_ of 18.40), Loch Rannoch (VAR_HD_ of 15.95), and Loch Shin (VAR_HD_ of 15.13) (Figure 1b). The lakes with low variability populations were dramatically less variable, for example, Loch More with VAR_HD_ of 0.87. The combined average variation for head depth (VAR_HD_) across all lakes was 7.24.

This pattern of adaptively relevant morphological variability was not an effect of larger population size within lakes. We found that allelic richness was not associated with any of the four morphological variables in any model (Table 1). Hence, neutral genetic diversity did not have a significant effect on the variability nor in predicting the extent of trophic morphology. In addition, abundance of Arctic charr did not have an effect on any of the morphological traits, indicating that intraspecific competition did not significantly influence the degree of variation or extent of adaptive morphology.

**Table 1:** Statistics for the best performing models (ecosystem size (=PC1), biotic community (=PC2), small, steep shore gradient, deep lakes (=PC3), genetic diversity, and charr abundance) and each morphological variable. Following model simplification, ecosystem size remained as the only significant parameter explaining morphology across all tests. Significant *p*‐values are shown in italics. Asterisks indicate significance levels, with *p < .05, **p < .01, and ***p < .001

Morphology	Best model	Estimate	*SE*	*t*‐Value	*p*‐Value	*R* ^2^
MEAN_HD_	MEAN_HD_ ~ PC1	−0.392	0.169	−2.317	.0297*	0.189
MIN_HD_	MIN_HD_ ~ PC1	−0.663	0.253	−2.621	.0153*	0.230
MAX_HD_	MAX_HD_ ~ PC1	1.306	0.443	2.949	.0070**	0.274
VAR_HD_	VAR_HD_ ~ PC1	1.524	0.296	5.156	<.0001***	0.536

### 3.3 Environment and the distribution of eco‐morphology

**Figure 2:**
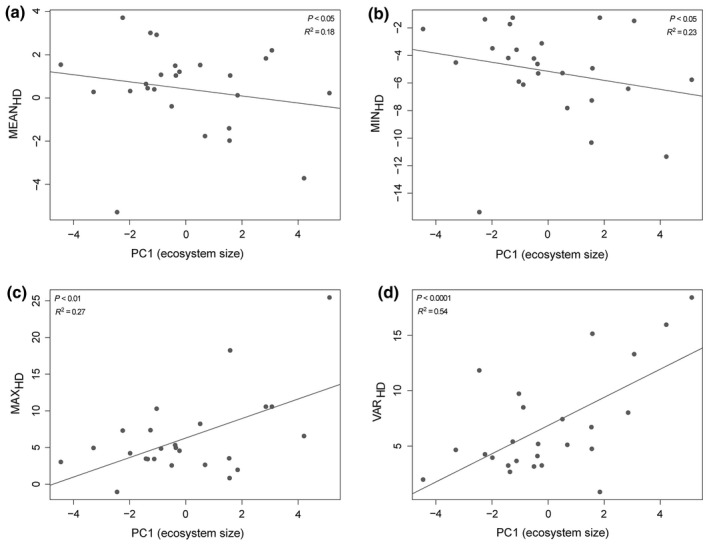
The relationship between Arctic charr morphological characteristics (a: MEAN_HD_, b: MIN_HD_, c: MAX_HD_, d: VAR_HD_) and lake environment parameters (ecosystem size) that were significantly associated in linear regressions

The average head morphology (MEAN_HD_) of an Arctic charr population was significantly negatively associated with ecosystem size (*p *=* *.030, *R* ^2^ **=* *0.19, coefficient* *=* *−0.392; Figure 2a). These results show that charr populations with more slender heads are more likely to be found in lakes with a larger ecosystem size (greater surface area, deep, voluminous lakes), relatively smaller littoral zone, and a more complex fish community (Table 1, Figure 2a).

The extremity of slender headedness (MIN_HD_) for a population showed a significant negative association with ecosystem size (PC1) in the multiple linear regressions (*p *=* *.015, *R* ^2^ **=* *0.23, coefficient* *=* *−0.663; Table 1; Figure 2b). In agreement with the regression approach, in the relative importance tests littoral zone area was also identified as the highest contributing factor (% of *R* ^2^ **=* *32.3%, Δ*R* ^2^ to next best factor* *=* *12.9%) (Figure S2). These results suggest that charr populations in shallower lakes are less extreme in MIN_HD_, while larger lakes tend to support individuals that are more extremely slender headed.

The extremity of deep headedness (MAX_HD_) of a population was positively associated with ecosystem size (*p *=* *.007, *R* ^2^ **=* *0.27, coefficient* *=* *1.306; Table 1; Figure 2c). This positive association remains as a trend even when excluding the most extreme MAXHD at highest ecosystem size (*p* = .266, *R* ^2^ = 0.06, coefficient = 0.416). Therefore, deep lakes with a greater surface area (larger ecosystem size) tend to support more extremely deep‐headed individuals. Similarly with the alternative relative importance approach, volume and surface area together explained more than half (59.6% of *R* ^2^) of the total variation in the correlation coefficient (*R* ^2^ **=* *0.58) between MAX_HD_ and all predictive lake parameters (Figure S2).

In summary, the average head depth of an Arctic charr population generally decreased to be more slender with larger ecosystem size (i.e., great surface area, deep, and voluminous lakes). The extremes of head depth (MIN_HD_ and MAX_HD_) of the Arctic charr populations increased with ecosystem size, and this pattern was stronger for MAX_HD_ compared to MIN_HD_. There was no significant association between MEAN_HD_, MIN_HD_, or MAX_HD_ with any of the principal components related to biotic community or to particularly small but deep lakes (Table 1).

### 3.4 Environment and the variability in eco‐morphology

The variation in head depth (VAR_HD_) of a charr population was highly significantly associated with ecosystem size (*p *<* *.0001, *R* ^2^ **=* *0.54, coefficient* *=* *1.524; Table 1; Figure 2d). Accordingly, with the relative importance approach volume and surface area explained more than 50% of the total *R* ^2^, with surface area being by far the largest contributor (32.1% of *R* ^2^) (Figure S2).

In summary, adaptively relevant morphological variation (VAR_HD_) in Arctic charr significantly increased with greater surface area and deeper lakes, or larger ecosystem size. Ecological parameters alone (PC2: fish community complexity, number of predators, and number of competing species) did not have a significant effect on variation in head depth, nor did genetic diversity (allelic richness) or population abundance (CPUE).

## 4 DISCUSSION

We found that the mean, minimum, and maximum head depth of Arctic charr varied greatly across populations in different lakes, as did variability (Figure 1b). In freshwater fishes, head depth is well established to be closely associated with alternative ecomorphologies (Adams et al., 2003; Liem, 1991; Seehausen & Wagner, 2014). Regarding mean and maximum head depths, a bulkier head and blunt‐snouted morphology is strongly associated with littoral foraging in Arctic charr. In contrast, limnetic or pelagic fishes are more active foragers, adapted to high swimming velocity and more elongated head shape to enhance the ability to capture evasive prey as reflected in the distribution of minimum and mean head depths (Adams & Huntingford, 2002a,b; Adams et al., 1998; Hooker et al., 2016; Klemetsen, Knudsen, Primicerio, & Amundsen, 2006; Kristjánsson et al., 2011).

Here, we found that the degree of head depth variation in an Arctic charr population was significantly predicted by ecosystem size. In order of importance, lakes that had a greater surface area, a greater volume, were deeper, supported a larger fish community, and had a proportionately smaller littoral zone (i.e., steeper slopes) supported populations of Arctic charr with a more variable head shape (Table 1, Figure 2d). This pattern was not driven by demographic effects associated with the size or abundance of a lake's Arctic charr population, as shown by the lack of any significant relationship between head depth measures or variability, population genetic diversity and CPUE.

Our results are consistent with the proposal that larger environments with consequently greater complexity support more niches; this allows local adaptations to the more diverse range of available resources and has been found consistently across aquatic and terrestrial habitats (Gavrilets & Losos, 2009; Post et al., 2000; Tews et al., 2004). For example, lakes with greater surface area have been shown to support a higher degree of ecomorphological differentiation in sticklebacks (McPhail, 1993; Schluter & McPhail, 1992), brook charr (Bertrand, Marcogliese, & Magnan, 2008), European whitefish (Siwertsson et al., 2010), and African cichlid species richness (Salzburger & Meyer, 2004; Wagner, Harmon, & Seehausen, 2014). Lake depth in particular has been associated with trophically relevant variability along a benthic–limnetic gradient in several freshwater fishes, including an increase in morphological variation or sympatric differentiation in European whitefish ecomorphs in Scandinavia (Hayden, Harrod, & Kahilainen, 2014; Siwertsson et al., 2010) and in the Alpine region (Vonlanthen et al., 2009), in Neotropical crater lake cichlids (Recknagel et al., 2014), and in African great lake cichlids (Wagner et al., 2012). While it has been hypothesized that deeper lakes also increase the potential for the evolution of trophically variable and polymorphic Arctic charr populations (Alekseyev et al., 2002; Hindar & Jonsson, 1982), this has not been tested robustly. This is the first study to support the significance of lake depth in predicting adaptive diversity in Arctic charr.

Another important component of ecological opportunity is the paucity of co‐existing species (Robinson et al., 1993; Vamosi, 2003), as this may open ecological space and reduce resource competition within lakes (Schluter, 2000). Here, we find no significant effect of fish community complexity and no effect of the number of competing species on Arctic charr population‐level morphological variation, mean, or extremes (Table 1; Table S2). In several species of postglacial fishes, including stickleback (Vamosi, 2003), pumpkinseed sunfish (Robinson et al., 1993), and brook charr (Bourke et al., 1999), it has been shown that if ecological niches are already filled by a different but ecologically similar species, this might impede the evolution of intraspecific variation and polymorphism. It has also been shown that despite occupying similar depth habitats in allopatry, Arctic charr, perch, and whitefish can co‐exist with each other, exhibit different trophic polymorphisms, and occupy different habitats in sympatry (Hayden et al., 2014; Sandlund et al., 2010). In contrast, other studies found a relatively low effect of competing species on the evolution of intraspecific variability and polymorphism (Eloranta, Nieminen, & Kahilainen, 2015; Recknagel et al., 2014; Svanbäck, Eklöv, Fransson, & Holmgren, 2008). Our study suggests that interspecific competition might not always limit the ability of a species to diversify, even in low productivity postglacial lakes; rather, the trophic morphology of an Arctic charr population depends primarily on the abiotic environmental characteristic of ecosystem size.

The link between environmental parameters and the degree of trophic variability and polymorphism is not well understood. Previous research in stickleback and whitefish suggests that disruptive selection is strongest when environmental contrasts are stark (Bolnick & Lau, 2008; Landry, Vincent, & Bernatchez, 2007). In the 30 lakes we studied here, the relative proportion of limnetic zone increases with lake size and depth and the relative proportion of littoral zone decreases. Small lakes are generally dominated by the littoral zone, providing suitable habitat and resources for benthic foraging. With an increase in lake size and depth, the relative proportion of the limnetic zone increases, opening a new niche for Arctic charr to exploit. This has also been shown for Scandinavian Arctic charr, which shift their diet to a more limnetic source with increasing lake size (Eloranta, Kahilainen, et al., 2015). The Arctic charr populations studied here are more variable, but also more extreme in their head depth in lakes that are larger and have a more complex habitat available or increased ecological opportunity. Because head depth measures reflect trophic niche use in charr (Adams & Huntingford, 2002b; Smith & Skúlason, 1996), our findings suggest that more specialized morphologies can be found in greater surface area and deeper lakes. In several geographically distant and unconnected lakes, the presence of specialized deep‐water ecomorphologies has been reported in Arctic charr (e.g., Alekseyev et al., 2002; Hindar & Jonsson, 1982; Hooker et al., 2016), strengthening the importance of environment for such morphologies to evolve.

The high degree of variation in head depth might result from phenotypically plastic individuals or genetic differentiation of divergent phenotypes within a lake's population. Most likely, a combination of both mechanisms is contributing to the observed phenotypic variation, as has been reported previously in Arctic charr (Jonsson & Jonsson, 2001; Adams & Huntingford, 2004). Depending on the time of colonization, the number of colonization events and other extrinsic factors, different lake populations will vary in the degree of how specialized and genetically divergent they are. While we have focused on the overall individual‐ and population‐level variation and have not assessed morphological specializations in these charr populations, variation may reflect subtle polymorphic divergences in sympatry (e.g., Adams et al., 1998; Garduño‐Paz et al., 2012). We did not find an effect of intraspecific competition and genetic diversity on the overall morphological variation; however, these factors likely have an impact on whether divergent ecomorphologies become initially established. For example, when conditions are stable over time (Taylor et al., 2006; Vonlanthen et al., 2012) and strong intraspecific competition prevails (Bolnick, 2004; Svanbäck & Bolnick, 2007), differentiation between individuals might become genetically fixed. This can be facilitated by differences in spawning time, spawning location, and habitat use that increases trophic variability, as has been evidenced repeatedly in Arctic charr (Adams et al., 1998, 2006; Jonsson & Jonsson, 2001). Our results suggest that environmental heterogeneity and consequently selection for phenotypic extremes in charr are strongest in deep lakes that are not dominated by littoral zone. This has the important implication that external context such as environment, rather than population‐specific intrinsic factors such as genetics (Elmer, 2016), determine the trophic variability and potential for adaptive diversification in these fishes.

## 5 CONCLUSION

We find that ecosystem size has a significant impact on adaptive morphological variation in an extensive diversification of Arctic charr. The most extreme head depths are found in larger lakes and the sympatric variation in head depth of a charr population significantly increased with lake ecosystem size. This adaptive morphological variation translates to high levels of extant diversity and may facilitate the formation of ecological specialists. The extent to which these diversifications are promoted by intrinsic factors such as genetic diversity vs. extrinsic factors such as environmental characteristics is still debated. Our findings suggest that ecological opportunities available through larger ecosystems (greater surface area, deeper, and more voluminous lakes) are the most significant component for this stereotypical diversification in a temperate radiation of freshwater fishes.

## CONFLICT OF INTEREST

None declared.
