# Supplementary material for: Ecosystem size predicts eco‐morphological variability in a postglacial diversification
Source: Ecol Evol. 2017 Jun 15;7(15):5560–70. doi: 10.1002/ece3.3013 (PMC5552947; doi:10.1002/ece3.3013)
Supplement: Supplementary file 1 [file ECE3-7-5560-s001.docx]

Table S1. Lakes in the study, number of charr sampled, and environmental parameters. The following syntopically occurring fish species were included in the ecological categories: (a) brown trout (*Salmo trutta*), (b) Atlantic salmon (*Salmo salar*), (c) roach (*Rutilus rutilus*), (d) rainbow trout (*Oncorhynchus mykiss*) and (e) powan (*Coregonus lavaretus*) were classified as competing species; (f) Northern pike (*Esox lucius*), (g) European eel (*Anguilla anguilla*), (h) European perch (*Perca fluviatilis*) and (a) brown trout (*Salmo trutta*) were classified as predators; all competitors and predators and (i) European flounder (*Platichthys flesus*), (j) brook lamprey (*Lampetra planeri*), (k) minnow (*Phoxinus phoxinus*), (l) three-spined stickleback (*Gasterosteus aculeatus*) were included in the community category. Abbreviations: DDM=Degrees Decimal Minutes; CPUE = catch per unit effort. Estimates of genetic diversity (measured as allelic richness) were extracted from Wilson et al. (2004).

| **ID** | **Lake name** | **Country** | **Latitude (DDM)** | **Longitude (DDM)** | **N charr** | **Volume (10^-6^ m^3^)** | **Max depth (m)** | **Mean depth (m)** | **Surface area (km^2^)** | **% Littoral** | **N community** | **N competitors** | **N predators** | **Genetic diversity** | **Abundance (CPUE)** |
| --- | --- | --- | --- | --- | --- | --- | --- | --- | --- | --- | --- | --- | --- | --- | --- |
| 1 | Uaine | Scotland | 57°31.022'N | 5°23.532'W | 23 | 0.02 | 3.05 | 1.07 | 0.03 | NA | 1[a] | 1[a] | 1[a] | 4 | NA |
| 2 | Coulin | Scotland | 57°32.648'N | 5°19.125'W | 13 | 2.55 | 14.94 | 5.57 | 0.39 | 84.6 | 1[a] | 1[a] | 1[a] | 5.8 | 1.12 |
| 3 | Mealt | Scotland | 57°36.346'N | 6°10.749'W | 40 | NA | NA | NA | 0.36 | NA | 1[l] | 0 | 0 | 2.5 | 18.20 |
| 4 | Fad | Ireland | 55°11.811'N | 7°9.182'W | 41 | NA | 15.3 | 4.4 | 0.14 | 83.5 | 0 | 0 | 0 | NA | 5.90 |
| 5 | Inagh | Ireland | 53°30.305'N | 9°44.481'W | 37 | 54.26 | 22 | 4.5 | 3.06 | 92.5 | 1[a] | 1[a] | 1[a] | 10.8 | 9.77 |
| 6 | Grilsta | Scotland | 60°15.030'N | 1°13.404'W | 18 | 8.72 | 22.56 | 9.57 | 0.9 | 48.4 | 2[al] | 1[a] | 1[a] | NA | NA |
| 7 | a' Ghriama | Scotland | 58°10.991'N | 4°44.118'W | 44 | 8.89 | 19.51 | 8.54 | 1.09 | 63.3 | 2[ab] | 2[ab] | 1[a] | 11.3 | 16.60 |
| 8 | Lee | Scotland | 56°54.284'N | 2°56.752'W | 22 | NA | 30 | NA | 0.88 | NA | 2[ag] | 1[a] | 2[ag] | 6 | 22.91 |
| 9 | Doine | Scotland | 56°20.410'N | 4°28.632'W | 34 | 5.55 | 19.81 | 10.1 | 0.72 | 45.8 | 3[agj] | 1[a] | 2[ag] | 5.5 | 24.55 |
| 10 | Clair | Scotland | 57°33.633'N | 5°20.673'W | 28 | 8.13 | 31.39 | 12.83 | 0.57 | 47.4 | 3[abk] | 2[ab] | 1[a] | 6.2 | 16.98 |
| 11 | Nalughraman | Ireland | 54°44.624'N | 8°31.803'W | 18 | 125.76 | 45 | 16.9 | 0.6 | 39.6 | 1[a] | 1[a] | 1[a] | 6.2 | 1.82 |
| 12 | Merkland | Scotland | 58°14.391'N | 4°44.492'W | 45 | 16.34 | 25.91 | 9.19 | 1.79 | 44.8 | 3[abk] | 2[ab] | 1[a] | 8.2 | 15.14 |
| 13 | Finn | Ireland | 54°51.613'N | 8°8.321'W | 24 | 51.07 | 23.8 | 7.4 | 1.15 | 73.9 | 4[abgk] | 2[ab] | 2[ag] | NA | 0.14 |
| 14 | Talla | Scotland | 55°28.743'N | 3°23.649'W | 57 | 12.54 | 22.25 | 10.58 | 1.22 | 30.2 | 4[abjl] | 2[ab] | 1[a] | NA | 3.63 |
| 15 | Voil | Scotland | 56°20.814'N | 4°25.587'W | 23 | 28.32 | 29.87 | 12.48 | 2.21 | 44.8 | 3[agj] | 1[a] | 2[ag] | 8.2 | 7.24 |
| 16 | a' Bhaid-Luachraich | Scotland | 57°49.166'N | 5°32.638'W | 34 | 13.76 | 43.59 | 10.36 | 1.29 | 56.2 | 4[abgk] | 2[ab] | 2[ag] | NA | 7.69 |
| 17 | Doo | Ireland | 53°39.059'N | 9°45.801'W | 34 | NA | NA | NA | 1.54 | NA | 1[a] | 1[a] | 1[a] | NA | 8.51 |
| 18 | Doon | Scotland | 55°15.111'N | 4°21.817'W | 33 | 42.96 | 30.48 | 8.14 | 5.28 | 52.4 | 3[abh] | 2[ab] | 2[ah | 13.8 | 2.95 |
| 19 | Langavat | Scotland | 58°2.299'N | 6°49.046'W | 58 | 67.62 | 29.87 | 7.56 | 8.94 | 64.6 | 3[abg] | 2[ab] | 2[ag | NA | 21.38 |
| 20 | Stack | Scotland | 58°20.142'N | 4°55.294'W | 39 | 27.98 | 32.92 | 10.95 | 2.58 | 62.4 | 5[abgil] | 2[ab] | 2[ag] | 11 | 3.89 |
| 21 | Lubnaig | Scotland | 56°17.709'N | 4°18.293'W | 40 | 32.39 | 44.5 | 13.04 | 2.36 | 55.5 | 5[abghj] | 2[ab] | 3[agh] | 7.2 | 14.79 |
| 22 | Dughaill | Scotland | 57°31.600'N | 5°10.534'W | 54 | 23.3 | 54.56 | 20.42 | 1.15 | 27.3 | 5[abgil] | 2[ab] | 2[ag] | NA | 1.09 |
| 23 | na Sealga | Scotland | 57°47.565'N | 5°18.475'W | 64 | 111.79 | 66.14 | 31.55 | 3.55 | 12.8 | 3[abg] | 2[ab] | 2[ag] | NA | 0.68 |
| 24 | Eck | Scotland | 56°5.072'N | 4°59.686'W | 22 | 67.42 | 42.37 | 15.29 | 4.35 | 35.2 | 7[abegjkl] | 3[abe] | 2[ag] | 4.2 | 7.08 |
| 25 | Shin | Scotland | 58°6.954'N | 4°34.469'W | 32 | 350.56 | 49.38 | 15.56 | 22.53 | 37.1 | 4[abgl] | 2[ab] | 2[ag] | 11.3 | 6.46 |
| 26 | More | Scotland | 58°17.419'N | 4°50.871'W | 21 | 139.55 | 96.32 | 38.35 | 3.44 | 20.1 | 3[abg] | 2[ab] | 2[ag] | 8 | 3.47 |
| 27 | Earn | Scotland | 56°23.010'N | 4°14.010'W | 47 | 408.36 | 87.48 | 42.01 | 10.13 | 9.6 | 5[abdkl] | 3[abd] | 1[a] | 6.7 | 5.89 |
| 28 | Maree | Scotland | 57°42.623'N | 5°31.835'W | 10 | 1091.3 | 111.86 | 38.1 | 28.6 | 29.9 | 4[abgk] | 2[ab] | 2[ag] | 13.7 | 3.31 |
| 29 | Rannoch | Scotland | 56°41.860'N | 4°13.695'W | 82 | 973.73 | 134.11 | 51.04 | 19.09 | 12.6 | 5[abfgh] | 2[ab] | 4[afgh] | 12.5 | 7.24 |
| 30 | Awe | Scotland | 56°17.965'N | 5°13.691'W | 54 | 1230.4 | 93.57 | 31.99 | 37.19 | 21.7 | 9[abcdfghkl] | 4[abcd] | 4[afgh] | 15.2 | 5.62 |

Table S2. Pairwise correlations between environmental lake parameters, with Pearson’s correlation coefficient (PCC) on the lower left diagonal and significance p-values on the upper right diagonal.

| **Parameter** | Volume | Max. depth | Mean depth | Surface | Littoral | N community | N predators | N competitors | CPUE |
| --- | --- | --- | --- | --- | --- | --- | --- | --- | --- |
| Volume |  | <0.001*** | <0.001*** | <0.001*** | 0.005** | 0.003** | 0.003** | 0.005** | 0.601 |
| Max. depth | 0.852 |  | <0.001*** | <0.001*** | <0.001*** | <0.001*** | 0.003** | 0.002** | 0.608 |
| Mean depth | 0.740 | 0.940 |  | <0.001*** | <0.001*** | <0.001*** | 0.003** | 0.005** | 0.619 |
| Surface | 0.879 | 0.704 | 0.566 |  | 0.007** | <0.001*** | <0.001*** | <0.001*** | 0.994 |
| Littoral zone | -0.549 | -0.761 | -0.887 | -0.438 |  | <0.001*** | 0.002** | 0.013* | 0.909 |
| N community | 0.451 | 0.537 | 0.507 | 0.551 | -0.555 |  | <0.001*** | <0.001*** | 0.652 |
| N predators | 0.493 | 0.525 | 0.483 | 0.577 | -0.509 | 0.829 |  | <0.001*** | 0.391 |
| N competitors | 0.528 | 0.574 | 0.464 | 0.582 | -0.355 | 0.671 | 0.398 |  | 0.794 |
| CPUE | -0.113 | -0.081 | -0.099 | 0.135 | 0.012 | 0.040 | 0.021 | -0.009 |  |

Table S3. Orthogonal data transformation with PCA. Loadings of lake parameters on the first three principal components (PCs) explaining 89.3% of the total variance (percentage of variance explained per PC shown in table). Bold values indicate a high importance of that parameter on the principal component (loadings > 0.3).

| **Parameter** | **PC1 (66.5%)** | **PC2 (12.8%)** | **PC3 (10.0%)** |
| --- | --- | --- | --- |
| Volume | **0.375** | 0.147 | **-0.449** |
| Surface | **0.359** | -0.126 | **-0.479** |
| Mean depth | **0.379** | **0.425** | 0.160 |
| Max depth | **0.401** | 0.283 | -0.084 |
| Littoral | **-0.334** | **-0.390** | **-0.480** |
| N community | **0.339** | **-0.490** | **0.352** |
| N competitors | **0.308** | **-0.364** | -0.251 |
| N predators | **0.324** | **-0.420** | **0.344** |

Figure S1. Principal component scores for all lakes with complete data (N=26). Directions for every lake parameter are indicated (e.g. lakes with a positive score on PC1 tend to have a large volume and a relatively smaller littoral zone). Parameters pointing in similar directions are positively correlated, while those pointing in opposing directions are negatively correlated (see Table S2 for correlation coefficients). Lake IDs are as indicated in Figure 1.


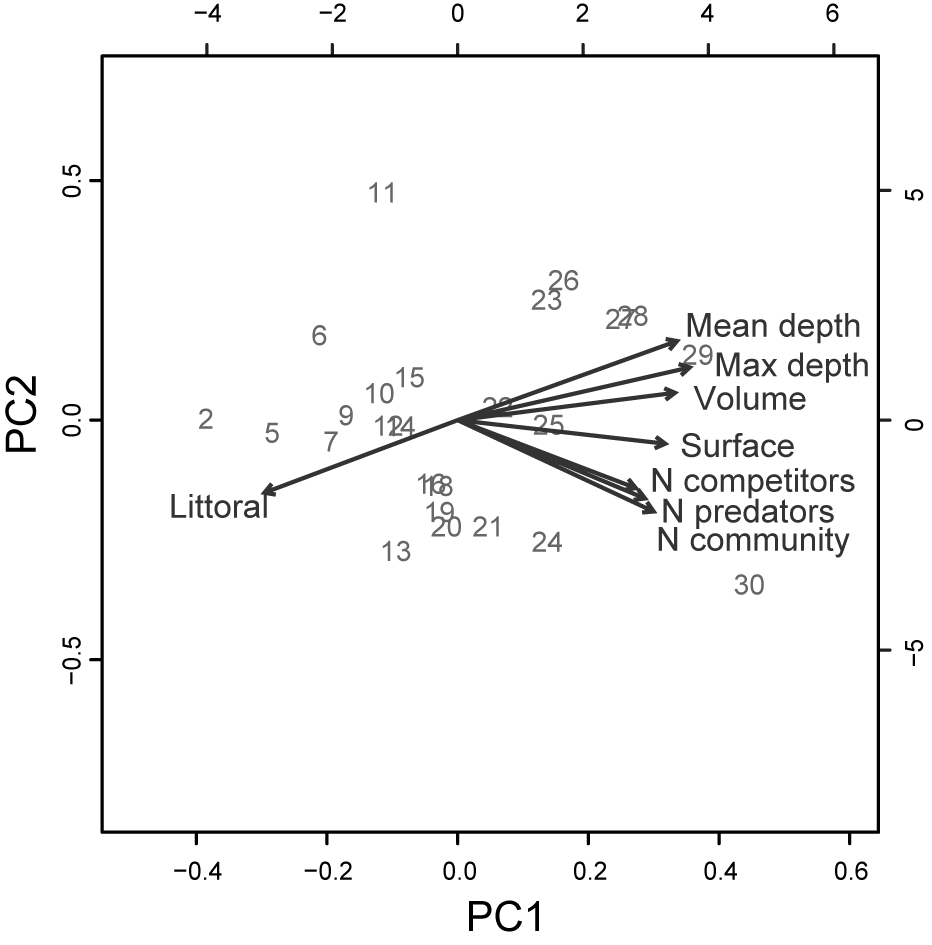


Figure S2. Relative importance test of all environmental lake parameters against head depth measures. Lines indicate 95% confidence intervals inferred from bootstrapping (N = 1000).
